# Virtual reality-based vestibular rehabilitation therapy in patients with acute unilateral vestibulopathy: a randomized controlled trial

**DOI:** 10.3389/fneur.2025.1519470

**Published:** 2025-01-28

**Authors:** Jae Woo Lee, Chul Young Yoon, Jae Ha Kim, Young Joon Seo, Tae Hoon Kong

**Affiliations:** ^1^Department of Otorhinolaryngology – Head and Neck Surgery, Yonsei University Wonju College of Medicine, Wonju, Republic of Korea; ^2^Department of Medical Informatics and Biostatistics, Yonsei University Wonju College of Medicine, Wonju, Republic of Korea; ^3^Research Institute of Hearing Enhancement, Yonsei University Wonju College of Medicine, Wonju, Republic of Korea

**Keywords:** vestibular rehabilitation, acute unilateral vestibulopathy, vestibular neuritis, virtual reality, randomized controlled trial

## Abstract

**Introduction:**

Conventional vestibular rehabilitation therapy (VRT) requires significant time and resources, especially for patients with low compliance, limiting its effectiveness and accurate assessment. Studies have shown VRT using virtual reality (VR) to be effective, with meta-analyses confirming its superiority over conventional methods. However, methodological variations in VR remain a limitation. This study aimed to assess the effects of VRT using head-mounted display (HMD) VR technology in patients with acute unilateral vestibulopathy (AUVP) and compare the outcomes with conventional VRT.

**Methods:**

We conducted a single-blinded randomized controlled trial with 60 AUVP patients randomly assigned to VR or control groups. The VR group received VRT via VR, while the control group underwent conventional VRT. Both groups followed individual home-based programs for 8 weeks and visited the clinic every 2 weeks. Subjective dizziness symptoms were evaluated using the visual analog scale (VAS), dizziness handicap inventory (DHI), and Activities-specific Balance Confidence (ABC) scale. Compliance with home-based programs was assessed on a 0–2-point scale based on responses in a booklet guide.

**Results:**

Seven patients were lost to follow-up, leaving 26 and 27 patients in the VR and control groups, respectively. The mean patient age was 56.91 ± 12.11 years; 22 men and 33 women were included. Two-way repeated measures analysis of variance showed significant improvement in both groups for DHI and ABC scores. However, changes in DHI, VAS, and compliance scores did not differ between groups. Improvement in the physical domain of the DHI and ABC scores was significantly faster in the VR group (*p* = 0.019 for DHI, *p* = 0.0020 for ABC).

**Discussion:**

VRT using VR technology showed comparable efficacy to conventional VRT in AUVP patients. The VR group demonstrated greater improvement in the physical domain of the DHI and ABC scales, indicating enhanced confidence in movement and reduced perception of physical handicap due to dizziness.

## 1 Introduction

Acute unilateral vestibulopathy (AUVP), also known as vestibular neuritis, is the sudden loss of peripheral vestibular system function, without any associated central neurological system disturbances or immediate hearing problems (1). AUVP is one of the top three common causes of peripheral vestibular disorders, along with benign paroxysmal positional vertigo (BPPV) and Meniere’s disease, with a reported annual incidence rate of 3.5 to 15.5 cases per 100,000 persons (1, 2). Short-term pharmacological intervention is recommended during the acute phase of AUVP, followed by targeted rehabilitation therapy for enhanced vertigo management (1, 3).

Vestibular rehabilitation therapy (VRT), which is an exercise-based treatment for advancing the natural process of vestibular compensation (4, 5), originated with the introduction of the Cawthorne–Cooksey exercises that were initially prescribed for patients with labyrinthine deficits following cranial trauma (6, 7). VRT is indicated in patients with acute and chronic non-progressive peripheral vestibulopathies (8). The main principle of VRT for promoting natural vestibular recovery is based on the vestibular compensation phenomenon, which is achieved by adaptation, habituation, and substitution exercises (3, 4).

Conventional VRT is performed in a clinical setting or as a home-based self-exercise following proper guidance and education, needs frequent in-person appointments, and is time-consuming and resource-intensive (5, 9). The amount of time and resources required for conventional VRT, particularly in patients with low compliance, are potential barriers to its incorporation resulting in further challenges in healthcare and therapeutic settings (10).

The use of virtual reality (VR) technology is widespread with reported applications in medicine, such as the generation of virtual environment using various types of display from video display to head-mounted display (HMD) (3, 11, 12). The effectiveness of VRT using VR technology has been reported in several studies (11, 13–16). A systematic review reported a standardized mean difference reduction of 1.13 in the Dizziness Handicap Inventory (DHI) scores in patients with peripheral vestibular disorders using adjunct VR interventions (14). However, methodological limitations were prevalent across studies due to high heterogeneity in experimental design, inclusion criteria, evidence-based clinical outcomes and the use of different VR interventions (13, 14, 16).

Thus, our study aimed to evaluate the efficacy and additional benefits of VRT using HMD assisted VR, and to provide further evidence on the utility of HMD assisted VR methods in VRT. We conducted a randomized controlled trial to compare the outcomes of VR-based VRT and conventional VRT in patients with AUVP.

## 2 Materials and methods

### 2.1 Participants

The authors performed a prospective single blinded randomized controlled trial in a single tertiary university hospital; 60 patients who were diagnosed with AUVP from July 2021 to June 2022 were included in this study. All the patients who participated in this study fulfilled the following inclusion criteria: (1) symptoms of acute onset vertigo lasting for at least 24 h; (2) findings of unidirectional peripheral vestibular hypofunction adhering to Alexander’s law upon physical examination, including the head impulse, nystagmus, test of skew (HINTS), and head shaking nystagmus; and (3) absence of central nervous system disorders resulting in vertigo and nystagmus. Neurotologic examinations, including spontaneous and gaze-evoked nystagmus, smooth pursuit and saccades, limb ataxia, and balance function, were performed in all the patients. Patients with isolated vertigo who demonstrated central ocular movements, limb ataxia, or severe imbalance underwent magnetic resonance imaging of the brain and those with any central lesions were excluded from the study. In addition, patients suspected of other peripheral vestibulopathies (other than AUVP), such as BPPV, Meniere’s disease, or bilateral vestibulopathy, were excluded. All the patients underwent a caloric test and a video head impulse test (vHIT); AUVP was defined as canal paresis (CP) greater than 24% in the caloric test or observed catch-up saccade with or without decreased gain in vHIT.

### 2.2 Study design and randomization

**Figure 1:**
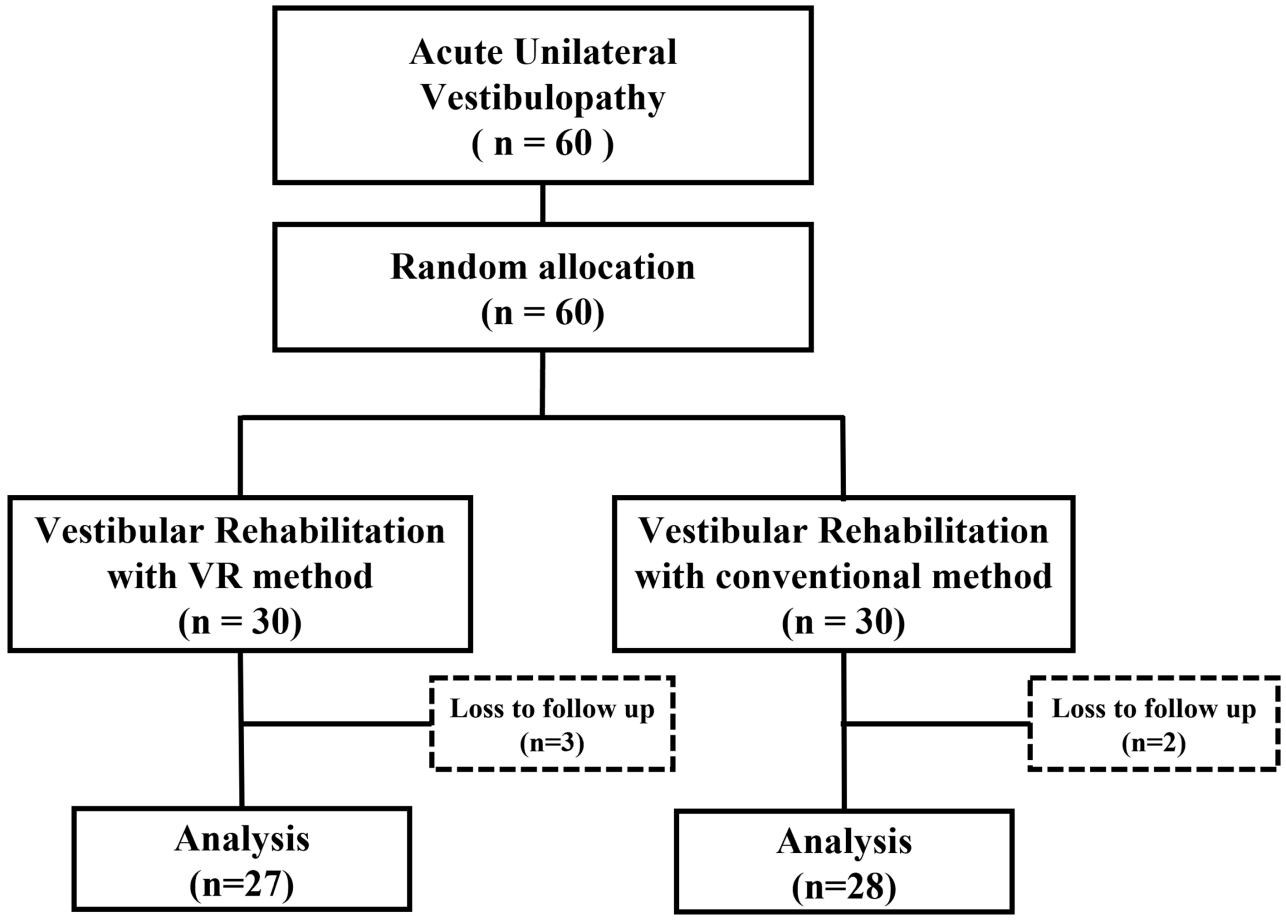
Study design.

Randomization was performed by assigning a random number to one of the two groups in advance; patients were randomly assigned to either the VR (*n* = 30) or control (*n* = 30) groups in the order of their enrollment in the study (Figure 1). The patients were not blinded to the treatment method they received. All the assessments and therapeutic interventions at the initial and follow-up sessions were performed by an independent otolaryngologist who was not part of the research team. The random numbering and assignment of patients to each group were performed in advance by an independent statistician, ensuring that the research team, including the PI, remained blinded. Data collection was carried out by an independent researcher and nurse (as described in the Acknowledgements). Throughout the data collection period, the research team members listed as authors were all blinded to the group assignments of the patients. Data analysis was conducted by the independent statistician. The PI was blinded to the vestibular rehabilitation received by the patient until the data analysis step, which marked the completion of the follow-up evaluations. The Institutional Review Board of our institute approved this study (CR320099). This study is registered with the Korean Clinical Research Information Service registry, which is one of the World Health Organization international clinical trial registry platforms (KCT0008480).

### 2.3 Study protocol

After assessing the eligibility based on the inclusion criteria and obtaining informed consent following a thorough explanation, all the participants were assigned to two groups via simple randomization. Patients initially underwent pharmacological therapy including the administration of sedative agents (antihistamines and/or diazepam) during the early acute phase when spontaneous nystagmus was observed in all the patients. The duration of pharmacologic sedative management ranged from a minimum of 2 days to a maximum of 5 days. They underwent VRT upon progression of AUVP when they were able to sit at the bedside as permitted by their health conditions.

**Figure 2:**
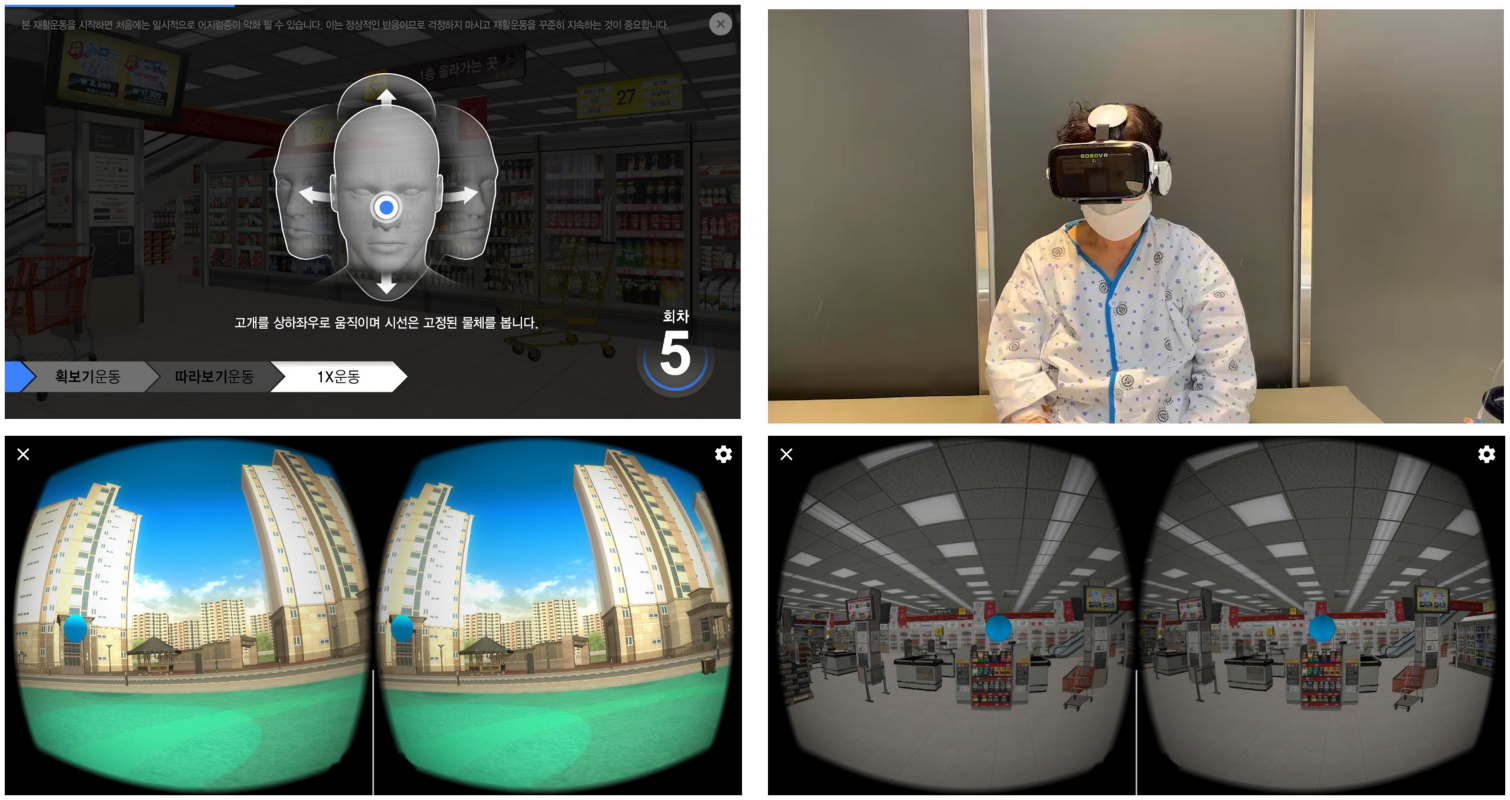
Vestibular rehabilitation therapy using virtual reality-based head mount display employed in this study.

The VR group underwent VRT with VR guidance (Figure 2), while the control group underwent VRT with booklet guidance. The VRT encompassed the purpose of VRT, precautions taken during execution, and exercises, including adaptation, habituation, substitution, balance, and gait. VRT incorporated in our study is based on the guidelines released by the Academy of Neurologic Physical Therapy of the American Physical Therapy Association and National Evidence-based Healthcare Collaborating Agency in Korea endorsed by relevant societies, including the Korean Society of Otorhinolaryngology – Head and Neck surgery, Korean Otologic Society, and Korean Balance Society.

The VR group used the following equipment: a 5.8″ display of an Android smartphone (Samsung Galaxy S9, Samsung Electronics, Seoul, Korea) placed in the HMD ‘BOBO VR Z4’ Headset (size length × width × depth = 194 × 117 × 127 mm, weight = 410 g, Xiaozhai, Shenzhen, China). The headset was adjusted every day by the patients themselves in terms of headband length, interpupillary distance, and focal depth of the spherical resin lens. At the beginning of the protocol, the patients were specifically trained in the clinic by an independent otolaryngologist with expertise in HMD implementation and blinded to the protocol.

The VR content primarily consisted of adaptation exercises and was structured into three difficulty levels: easy, normal, and hard. At each level, the object the patient needed to focus on through VR remained the same (a blue ball as shown in Figure 2), but the background varied. The easy level had a simple background (e.g., a room), the normal level had a slightly more complex background (e.g., an apartment complex park), and the hard level had a complex background (e.g., inside a large supermarket) (Figure 2). Patients were instructed to maintain a speed at which they could perform 15 back-and-forth head movements. As they felt less dizziness during performing the current level of vestibular rehabilitation exercises, they were instructed to perform the movements more quickly. Patients started at the easy level and were directed to progress to the normal and hard levels if they did not feel dizzy.All the participants were advised to perform the VRT exercise three times a day – in the morning, afternoon, and evening – and were recommended to engage in each session for at least 20 min.

All the participants underwent baseline assessments of dizziness and balance perception using the DHI and Activities-specific Balance Confidence (ABC) scale before starting VRT. They visited the hospital every 2 weeks to receive guidance on vestibular rehabilitation. During each visit, their improvement in AVUP was evaluated using the DHI, ABC, VAS, and the compliance scores. Based on these evaluations, they received personalized guidance on vestibular rehabilitation exercises, and performed at home for the next 2 weeks. This routine continued for a total of 8 weeks. Additionally, patients recorded their subjective dizziness changes daily on a visual analog scale (VAS) ranging from 0 to 10 (0 indicates no dizziness and 10 indicates marked dizziness) and marked their adherence to VRT on a 0–2-point scale each day to assess the compliance. Both groups were given a paper to record VAS and compliance, which they could fill out daily. Patients brought this record to the hospital every 2 weeks, and the VAS and compliance they recorded daily was collected at that time. The daily recorded VAS were averaged for each two-week period upon the participants’ biweekly visits, and the compliance score was assigned as follows: patients were instructed to record their performance for each session—morning, afternoon, and evening—by marking an “O” if they completed 20 min or more, a triangle (△) if they completed only part of the session of 20 min, and an “X” if they were unable to perform the session at all. They were assigned scores of 2 points for an “O,” 1 point for a triangle, and 0 points for an “X.” A maximum daily score of 6 points was evaluated every 2 weeks (14 days) and converted to a percentage, with 84 points representing 100%.

### 2.4 Statistical analysis

We performed the Shapiro–Wilk test to assess whether the continuous variables followed a normal distribution. Since all continuous variables were normally distributed, differences between the VR and control groups were compared using the student’s t-test for unpaired data. Chi-square test or Fisher’s exact test were performed to compare the categorical variables between the two groups. To compare the improvement in dizziness between the two groups, repeated measures analysis of variance was performed to compare the DHI, ABC scales, and VAS scores and compliance of VRT between both groups. Statistical analyses were performed using SAS 9.4 (SAS Institute Inc., Cary, NC, United States). For all the statistical tests, a *p*-value of <0.05 was considered statistically significant.

## 3 Results

Among the 60 patients included in the trial, 3 and 2 patients of the VR and control groups, were lost to follow-up, leaving 27 and 28 patients, respectively (Figure 1). The mean age of the total 55 patients was 56.91 ± 12.11 years; 22 (40.0%) males and 33 (60.0%) females were included in the study. AUVP was present on the left and right side in 21 (38.2%) and 34 (61.8%) patients, respectively. The average CP value of the caloric test in AUVP was 37.71, and the average directional preponderance (DP) value was 16.75. On the vHIT, covert or overt catch-up saccades were observed on one side in all the patients, with an average reduced gain value of 0.63 (Table 1). No significant difference was observed in the age, sex, affected side, and CP and DP values. In the vHIT, there was a significant difference between the gain values of both groups (*p* = 0.045).

**Table 1:** Clinical and demographic characteristics of the participants.

	Total (n = 55)	VR group (n = 27)	Control group (n = 28)	*p*-value
Age (days, mean ± SD)	56.91 ± 12.11	58.56 ± 13.01	55.32 ± 11.18	0.327
Sex (Male: Female)		11:16 (40.7%: 59.3%)	11:17 (39.3%: 60.7%)	1.000
Affected side (Right: Left)		9:18 (33.3%: 66.7%)	12:16 (42.9%: 57.1%)	0.582
Canal paresis (%, mean ± SD)	37.71 ± 25.43	43.53 ± 23.82	31.39 ± 26.10	0.092
Directional preponderance (%, mean ± SD)	16.75 ± 13.21	19.85 ± 13.37	13.40 ± 12.43	0.084
Minimum gain in vHIT (mean ± SD)	0.63 ± 0.24	0.54 ± 0.26	0.68 ± 0.21	0.045*

Values are presented as mean ± SD for continuous variables, and numbers as percentages for categorical variables. *p < 0.05. SD, standard deviation; vHIT, video head impulse test.

At baseline, before starting VRT, no significant differences were observed in the DHI and ABC scores, and the functional, emotional, and physical subscales of the DHI between both the groups (Table 2).

**Table 2:** Baseline scores of DHI (Dizziness Handicap Inventory) and ABC (Activities-specific Balance Confidence) questionnaires among participants.

	Total (*n* = 53)	VR group (*n* = 27)	Control group (*n* = 28)	*p*-value
DHI – Total (score, mean ± SD)	48.84 ± 22.96	51.11 ± 21.95	46.64 ± 24.09	0.476
DHI – Functional (score, mean ± SD)	20.73 ± 10.17	22.15 ± 9.91	19.36 ± 10.41	0.313
DHI – Emotional (score, mean ± SD)	14.73 ± 9.79	14.52 ± 8.60	14.93 ± 10.96	0.878
DHI – Physical (score, mean ± SD)	13.35 ± 5.85	14.37 ± 5.35	12.36 ± 6.23	0.205
ABC (score, mean ± SD)	48.23 ± 27.30	43.82 ± 28.17	52.46 ± 26.23	0.244

Values are presented as mean ± SD for the continuous variables. DHI, Dizziness Handicap Inventory; ABC, Activities-specific Balance Confidence; VR, virtual reality; SD, standard deviation.

**Figure 3:**
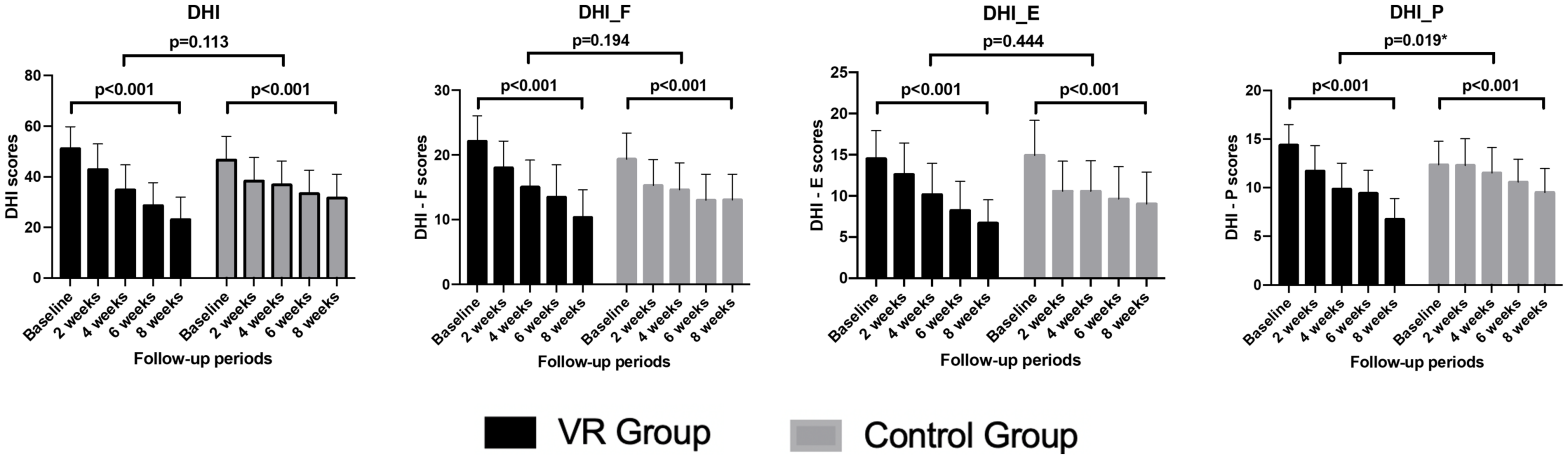
Comparison of the changes in the subscores and total score of the DHI over the 8-week vestibular rehabilitation therapy period. DHI, dizziness handicap inventory; DHI_F, functional domain of the dizziness handicap inventory; DHI_E, emotional domain of the dizziness handicap inventory, DHI_P; physical domain of the dizziness handicap inventory.

As VRT progressed, a significant decrease in the DHI scores was observed in both groups, with no significant difference in the pattern of reduction between the groups. Considering the DHI subscales, no significant difference was observed in the patterns of decrease in the functional and emotional subscales between the groups. However, the reduction was more rapid and greater in the VR group compared to the control group in the physical subscale, indicating a significant difference (Figure 3).

**Figure 4:**
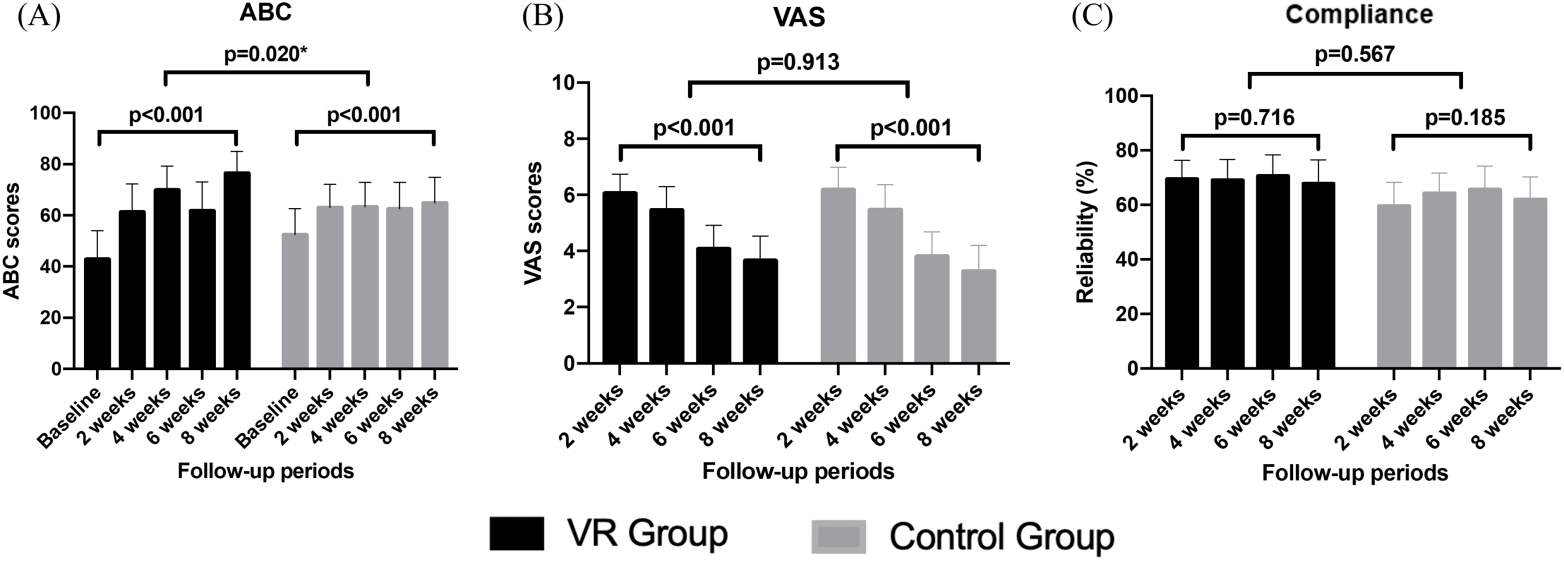
Comparison of changes in the ABC scale **(A)**, VAS **(B)**, and compliance scores **(C)** over the 8-week vestibular rehabilitation therapy period.

The ABC scale showed significant improvement in both groups with VRT progression. The degree of improvement was significantly greater and faster in the VR group compared to that in the control group (Figure 4A). The subjective symptom of vertigo, assessed using the VAS, significantly decreased in both groups. However, no significant difference in the extent of this decrease was observed between the groups. Additionally, VRT compliance did not show significant changes during the treatment and did not vary between both groups (Figures 4B,C).

Table 3 presents the comparison of the compliance at two-week intervals; no significant differences were observed between the two groups at each time point. During the first 2 weeks, a difference of approximately 10 points was observed in the compliance between both groups; however, this finding was not statistically significant (*p* = 0.071).

**Table 3:** Comparison of the compliance scores between two groups at bi-weekly visits over the study period.

	Total (*n* = 55)	VR group (*n* = 27)	Control group (*n* = 28)	*p*-value
Compliance – 2nd week (score, mean ± SD)	64.51 ± 19.93	69.54 ± 16.81	59.66 ± 21.75	0.071
Compliance – 4th week (score, mean ± SD)	66.63 ± 18.85	69.07 ± 18.95	64.27 ± 18.81	0.359
Compliance – 6th week (score, mean ± SD)	68.07 ± 20.59	70.60 ± 19.22	65.64 ± 21.91	0.386
Compliance – 8th week (score, mean ± SD)	64.84 ± 21.33	67.79 ± 21.61	61.99 ± 21.05	0.327

Values are presented as mean ± SD for continuous variables. VR, virtual reality; SD, standard deviation.

## 4 Discussion

This study assesses the effectiveness of VR-assisted VRT during the acute phase of patients diagnosed with AUVP. To our knowledge, our study is the first to investigate the effectiveness of VR-assisted VRT versus conventional VRT in the acute phase of fixed non-compensated AUVP through a single-blinded randomized controlled trial.

In our study, the DHI scores significantly decreased in both groups, indicating the effectiveness of VR-assisted VRT, which was comparable to that of conventional VRT, in improving dizziness during the early acute phase of AUVP. Notably, no significant difference was observed in the improvement of the emotional and functional DHI subscales between both groups; however, a significant difference was observed in the physical subscale. Unlike conventional VRT, which requires learning and practice and is performed by the patients themselves without feedback, VR-assisted VRT requires less effort for learning and immediate postural visual feedback is provided through VR with HMD. This helps patients maintain appropriate postural stability during exercise and sparks interest, thereby enhancing performance and improving physical activity. This is supported by the significantly greater improvement in the ABC scale scores in the VR group, indicating reduced dizziness-related activity limitations. Meldrum et al. (17) also reported similar improvement in dizziness but with higher compliance using Nintendo Wii^®^ video games assisted VRT compared to that using conventional VRT. This underscores that VR-assisted VRT, which provides immediate feedback on movements without the need for specialized training, can stimulate interest in vestibular rehabilitation exercises and encourage more active participation (17).

We measured the VAS scores to compare the degree of subjective symptom improvement in vertigo among participants following VRT. The decreasing trend of the VAS score by 2 weeks was similar to the reduced DHI scores, indicating improvement. As described in the Methods section, patients were instructed to either perform the same level of exercise at a faster pace or progress to the next level if they experienced less dizziness while performing the current level. During biweekly visits, the trend of VAS reduction could influence the decision to progress to the next stage of VRT. In our study, there was no significant difference in the trend of VAS reduction between the two groups, and the final exercise level achieved over 8 weeks was identical in both groups.

In our study, we incorporated a daily 0–2-point scale for measuring VRT compliance. No difference was observed in the compliance between the two groups, indicating that VRT was applied consistently across both groups regardless of compliance. We expected significantly better compliance in the VR group than in the control group in the first 2 weeks owing to the incorporation of adaptation exercises using a VR-based HMD. However, this difference was not statistically significant in our study. Previous studies reported that VR technology, including video games, in VRT showed better compliance (14, 17, 18). In a study on the effects of VR in VRT for patients with persistent postural-perceptual dizziness, compliance was maintained by conducting follow-up visits every 2 weeks during an 8-week tracking period (19). Thus, follow-up visits every 2 weeks can be considered a practical method for maintaining compliance in patients based on the accessibility to healthcare facilities and scope of the institutions (10).

Our study had several limitations. First, the HMD-only based VR technology used in our study can only be applied to adaptation exercises for vestibulo-ocular reflex training in initial VRT, making it challenging to elucidate the overall effect of VR in VRT. To perform substitution or habituation exercises aimed at recovering postural balance performance using VR, additional posture measurement equipment, such as a balance board or walking platform, is warranted. Therefore, further research is necessary for developing posture measurement equipment that can be integrated with our VR system. Second, all the participants in our study were only evaluated using subjective scales, such as questionnaires or the VAS, for the improvement of dizziness during VRT in AUVP. Objective methods, such as dynamic or static posturography, were not incorporated, and the effect of VR therapy was not measured according to the compensatory strategies for AUVP. Hence, the authors could not objectively support the improvement in dizziness or postural balance perception attributed to AUVP. As a previous study with adequate compensatory strategies in patients with AUVP showed multidimensional improvements, including balance performance, lower perceived handicap, and lower anxiety and depression, future studies are warranted to measure the effects of central compensation for vestibular impairment, multisensory substitution, and habituation in VRT using VR technology in a more comprehensive manner (20). Third, the 0–2-point compliance scale used in our study may have influenced the results. Participants recorded triangle for not completing the full 20 min of exercise in each VRT session likely had a wide range of actual practice times, ranging from more than 1 min to less than 20 min. The 0–2-point scale was too narrow to accurately reflect compliance, and using the actual duration of exercise (in minutes) as a compliance score might have provided a more precise measure of compliance. Furthermore, the study relied on voluntary participation, and participants were required to attend biweekly follow-up visits. As a result, the compliance observed in the study participants may differ from the actual compliance of patients performing VRT (in any methods) outside of a research setting. Additionally, our sample size may not have been sufficient to adequately compare compliance between groups. Future studies is needed to compare accurate compliance between conventional VRT and VR-based VRT.

In conclusion, VRT using VR-based HMD demonstrated comparable efficacy to that of conventional VRT in managing patients with AUVP over an 8-week period. Notably, improvements in dizziness, particularly in the physical domain of the DHI, which reflects physical self-perceived handicap, and in the ABC scale, which reflects specific complaints concerning daily activities, were significantly more pronounced in the VR group compared to that in the control group. Additionally, biweekly follow-ups over the course of the 8-week VRT demonstrated similar levels of compliance in both the VR and control groups.

## Data Availability

The datasets presented in this article are not readily available because data from this study are available upon reasonable request, subject to ethical restrictions and approval by the Institutional Review Board (IRB). Requests to access the datasets should be directed to Tae Hoon Kong, cochlear84@yonsei.ac.kr.
